# Editorial: Tinnitus in relation to auditory processing: unravelling complex relationships

**DOI:** 10.3389/fnins.2026.1814945

**Published:** 2026-10-19

**Authors:** Takwa Gabr, Prashanth Prabhu, Haúla Haider

**Affiliations:** 1 Department of Otorhinolaryngology, Kafrelsheikh University, Kafr El-Shaikh, Egypt; 2 Department of Otorhinolaryngology, Faculty of Medicine, University of Sadat City, Sadat City, Egypt; 3 All India Institute of Speech and Hearing (AIISH), Mysore, India; 4 CUF Infante Santo Hospital, Lisbon, Portugal

**Keywords:** central auditory processing, listening effort, repetitive transcranial magnetic stimulation (rTMS), tinnitus, tinnitus definition

## Introduction

Tinnitus is a perceptual phenomenon impacting 10% to 15% of the global population. In majority patients, it significantly undermines their quality of life and their emotional stability. In this Research Topic in Frontiers in Neuroscience, titled “*Tinnitus in relation to auditory processing: unravelling complex relationships*,” we aimed to discuss different aspects of tinnitus from theories, clinical inquiries to advanced diagnostic procedures to address the complex tinnitus dilemma. This topic included six articles highlighting different perspectives of tinnitus from definition, impact on life, diagnosis, and management.

## Key themes and insights

In his article titled “*Defining tinnitus: a socratic and epistemological inquiry*” Aazh tried to find a definition of tinnitus using a Socratic and epistemological inquiry away from the traditional definition of being phantom without an external source (Jastreboff, 1990, 1995) or meaning (Noreña et al., 2021). The author argues that these formulations are insufficient as they fail to distinguish tinnitus from other internal auditory experiences, such as the sounds heard in dreams, or auditory hallucinations. In his trial to find a definition of tinnitus, the author pursued what is shared by all cases of tinnitus and belongs to nothing else. He was derived by several factors: *first*, the wide prevalence rates of tinnitus that vary from 5% to 37% indicate an inconsistent definition of tinnitus (Jarach et al., 2022). *Second*, the frequent confusion between tinnitus descriptions and its definitions. *Third*, how to distinguish the definition of tinnitus itself from the meanings individuals may attribute to it (Davis and Refaie, 2000). Using the Socratic approach, Aazh developed a working definition of tinnitus as “the consciously recognized experience of a non-semantic, sound or sound-like event in wakeful consciousness that persists beyond fleeting moments and becomes experientially salient.” The main aim of such formulation was to treat tinnitus as a hypothesis and to avoid imposing a conceptual uniformity on a heterogeneous experience.

Another article investigated the nature of tinnitus in our issue, provided by Barker et al., examined the Zwicker tone (ZT) to understand the nature of tinnitus perception. ZT is an auditory illusion tone experienced by about 42% of the normal hearing population after exposure to “notched noise.” Interestingly, ZTs can be perceived by about 95% of tinnitus cases (Parra and Pearlmutter, 2007). It is commonly recorded in response to the presentation of notched noise (NN), where a faint, quickly decaying pure tone, the frequency of which falls within the range of the notch, is noticed (Schilling et al., 2023). Both tinnitus and the Zwicker Tone have common features like being illusory sounds associated with a spectral contrast (tinnitus is often linked to hearing loss, while ZT is triggered by a notch in a low-frequency band-pass noise), and both percepts fall within the unstimulated frequency region (hearing loss region for tonal tinnitus) and within the notch for ZTs. Additionally, the exact mechanism that creates ZTs, as in the case of tinnitus, and where in the brain it originates is still a matter of debate. The study highlighted the importance of using ZTs as a controllable laboratory model for tinnitus to understand the underlying neural mechanisms of chronic tinnitus.

Another study in this issue named “*Sex-specific alterations of cerebral blood flow in subjective tinnitus patients: an arterial spin labeling study*” where Zhang et al. examined the sex-specific alterations of cerebral blood flow (CBF) in chronic subjective tinnitus patients using arterial spin labeling (ASL). CBF is considered a direct index of neuronal activity closely related to the metabolic needs of the neural tissue. ASL is a non-invasive, magnetic resonance perfusion imaging technique used for quantification of the whole-brain CBF (Zimmerman et al., 2021) and used as an indicator of changes in the central neuronal activity (Chen et al., 2022). In this study, tinnitus patients showed relative hypoperfusion at the inferior frontal gyrus, superior temporal gyrus, insula, and opercular regions; with relative hyperperfusion in post-central gyrus, inferior parietal lobule, angular gyrus, supramarginal gyrus, and superior parietal lobule. The CBF in opercular inferior frontal gyrus was positively correlated with tinnitus loudness. Male tinnitus patients showed relative greater hyperperfusion in frontal and precentral cortices, while the female patients showed stronger increases in parietal regions. In healthy controls, males showed greater perfusion in medial prefrontal regions, and females showed stronger activity in sensorimotor and associative cortices. This study suggested that chronic tinnitus is associated with a widespread CBF alterations in both auditory, cognitive–emotional and attentional networks. The presence of specific neuropathological mechanisms in those patients is suggested by the sexually dimorphic perfusion patterns. It also supports the promising use of ASL perfusion imaging as a tool for assessing tinnitus patients.

Using a multidimensional approach, Giliberto et al. investigated the cognitive and emotional burden of tinnitus in their article titled “*Listening effort and stress in tinnitus: a multidimensional approach*.” In their study, they compared 24 patients with 23 controls during a listening task (audiobook excerpts) presented at different signal-to-noise ratios (0, +5, and +10 dB). They also combined subjective measures [such as Tinnitus Handicap Inventory (THI), Tinnitus Questionnaire (TQ12), State-Trait Anxiety Inventory (STAI), and a hyperacusis questionnaire] with objective markers (EEG, heart rate, skin conductance, and salivary stress hormones) to assess potential changes in biochemical markers associated with stress and anxiety. Tinnitus patients showed a diminished speech comprehension particularly in quiet environments and at moderate noise levels (SNR10 and SNR5). This potential disruption in the cognitive processes in those patients aligns with the possible tinnitus interference of central auditory processing (Tzounopoulos et al., 2019). Interestingly, this decline was absent under high-noise conditions (SNR0), suggesting the possible masking effect of environmental noise on tinnitus. The alpha-amylase levels were higher among all tinnitus patients regardless of tinnitus status indicating an acute stress response triggered by noise exposure through activating the sympathetic system (Ali and Nater, 2020). Females showed more tinnitus perception and difficulty in moderate and high-noise environments (SNR0 and SNR5) than males suggesting their higher susceptibility to tinnitus and their increased emotional distress (Simoes et al., 2021). These finding suggests the influence of social and cultural expectations on the individuals' experiences where women are more likely to align their responses with perceived social norms and underreport their negative experiences or overstating the positive ones (Ng et al., 2024). The complex correlation between STAI scores, questionnaire scores, salivary amylase and cortisol levels, and heart rate (HR) across various experimental phases emphasize the need to shift toward multimodal, personalized, and gender-sensitive approaches in the assessment and management of tinnitus.

This Research Topic addresses the management of tinnitus. In their meta-analysis titled “*Efficacy of repetitive transcranial magnetic stimulation for subjective chronic tinnitus: a randomized controlled trial meta-analysis*” He et al. examined the use of rTMS as a non-invasive technique for modulating neural activity in tinnitus patients in conjunction with both THI and VAS. The study showed lower tinnitus loudness immediately and for 1-month post-treatment. However, this effect diminishes gradually to reach uncertain level at6months indicating that the duration and the frequency of rTMS treatment protocol need to be refined to have more prolonged relief.

Another article addressed the efficacy of music therapy in chronic patients using the “short-course acoustic test.” In their article, Ni et al., examined 515 tinnitus patients using extended high-frequency audiometry, psychophysical assessment of tinnitus regarding frequency and intensity, THI, VAS and Hospital Anxiety and Depression Scale (HADS). The study included a Short-Course Acoustic Test where tinnitus patients underwent a 15-min session of Tinnitus Multielement Integration Sound Therapy (T-MIST), that used a mixture of narrowband noise (I-tone), music (Transfocus), and natural environment sounds (D-sound) designed to mask the tinnitus. Based on their VAS scores done immediately after T-MIST, patients were categorized into three groups: Tinnitus Disappeared Group (0 VAS score), Tinnitus Relieved Group: Noticeable reduction in VAS, but > 0 and Tinnitus Unchanged Group (same VAS score). Through an AI-application “Tinnitus Assistant” all individuals' data of patients, their test results and questionnaire scores were used to create a personalized sound profile (music) that can be used to suppress tinnitus. This music was edited to be 180° out of phase with the frequency of patient's tinnitus for reduce cortical perception of tinnitus. Patients were instructed to listen for more than 2 h daily (in total) for a least 30 min at a time in a quiet environment for 10 months followed by assessment using VAS, THI, HADS-A. The treatment efficacy rate was higher in the tinnitus disappeared group than that of other groups. Findings of this study suggested that CMT can, to some extent, be effective in patients with chronic subjective tinnitus with a promising role as a guide during clinical therapy.

## Future directions

This issue highlighted the importance of future direction in customizing tinnitus diagnosis and management. It also highlighted the importance of considering the gender impact on tinnitus pathogenesis. The connection between tinnitus and cognition should be targeted more in future research specially those using advanced imaging techniques and address the co-morbidity between tinnitus and the functional impairment in the central nervous system.

## Conclusion

This Research Topic in this issue of Frontiers in Neuroscience emphasizes the complex nature of tinnitus where its profound personal experience requires multimodal approaches. A more comprehensive definition of tinnitus is required with more concentration of the individual factors specially the physiological difference between males and females in brain perfusion and neural network that requires a new direction in the diagnostic tools. By understanding the unique neural signatures and cognitive burdens of each patient—whether influenced by their sex, their stress response, or their physiological reaction to sound—we can move away from standardized solutions. The relationships between tinnitus and auditory processing are indeed complex, but through this integrated research, we are beginning to unravel the threads.
